# “How did I get here?”: a qualitative life-course analysis of Australian women’s experiences of elder abuse

**DOI:** 10.1093/geront/gnag122

**Published:** 2026-06-05

**Authors:** Bianca Brijnath, Rachel Muoio, Jake Najman, Shelby Marrington, Alexandra Clavarino, Tara Renae McGee, Anna Gilbard

**Affiliations:** School of Humanities and Social Sciences, La Trobe University, Melbourne, Victoria, Australia; Division of Social Gerontology, National Ageing Research Institute, Melbourne, Victoria, Australia; School of Public Health, University of Queensland, Brisbane, Queensland, Australia; School of Public Health, University of Queensland, Brisbane, Queensland, Australia; School of Public Health, University of Queensland, Brisbane, Queensland, Australia; School of Criminology and Criminal Justice, Griffith University, Mount Gravatt, Queensland, Australia; Family & Disability Services, UnitingCare Community, Toombul, Queensland, Australia

**Keywords:** Elder abuse, Life-course, Lived experience, Poly victimization, Poverty

## Abstract

**Background and Objectives:**

We applied a life-course perspective to understand how older women’s reported experiences of serious elder abuse were shaped by a history of violence and poverty.

**Research Design and Methods:**

17 participants (mean age 65.8, SD 4.5) self-reporting experiencing elder abuse within the past 12 months were purposively recruited from the longitudinal Mater-University of Queensland Study of Pregnancy cohort. They participated in a 1-hour semi-structured qualitative interview between April and November 2024 and their data were analyzed using a thematic approach.

**Results:**

Participants were relatively healthy, cognitively unimpaired, and mainly from a low-income background (13, 76.5%). Elder abuse was part of a lifelong continuum of family violence beginning in childhood or midlife, with poly-victimization common and multifaceted. Poor health and advancing age compounded participant’s vulnerability, while cumulative economic disadvantage was gendered and heightened women’s risk in later life. Finally, violent and misogynistic media content, a growing intergenerational wealth gap, and gendered ageism were seen as contextual factors deepening intergenerational divides and eroding familial cohesion.

**Discussion and Implications:**

This study is the first in Australia to examine elder abuse through a life-course lens, drawing directly on older women’s lived accounts. The findings demonstrate how interlocking patterns of dependence, declining health, and cumulative inequality converge in later life. Viewing elder abuse through this perspective moves attention beyond isolated incidents to the broader patterns that shape vulnerability, informing prevention and policy strategies that tackle the structural and relational determinants of abuse across the lifespan.

## Background and objectives

About 1 in 6, or 68 million older women experience abuse worldwide (Yon et al., 2019). Elder abuse victimization has been linked to declines in physical and mental health, higher rates of substance use, greater social isolation, and an elevated risk of suicidal behavior (Acierno et al., 2017; Folorunsho & Okyere, 2025; Yunus et al., 2019). Exploring elder abuse through a life-course perspective can further our understandings of factors contributing to elder abuse and individual vulnerabilities, by situating individual experiences within broader temporal, social, and historical contexts, and by examining the way in which past experiences may cumulatively shape risk (Dannefer, 2003; Herrenkohl et al., 2022; McDonald & Thomas, 2013). In this article, we applied a life-course perspective to understand how older women’s reported experiences of serious elder abuse were shaped by a history of violence and poverty.

### A life-course perspective

Life-course studies of elder abuse victimization show that many older adults who experience abuse have histories of trauma, family conflict, or disadvantage, which can shape their vulnerability to elder abuse in later life (Awuviry-Newton et al., 2025; Easton & Kong, 2021; Herrenkohl et al., 2022). Individuals who have experienced abuse in childhood or as adults are more likely to experience elder abuse in later life. For instance, in a large survey, having a history of abuse in childhood was a significant predictor of elder abuse in later life, even after accounting for other risk factors (Herrenkohl et al., 2021). Similarly, exposure to intimate partner violence in midlife can increase the risk as much as threefold and eightfold for financial and psychological abuse in later life (Wang & Dong, 2019).

Research also shows that poly-victimization, i.e., being subjected to multiple forms of abuse, is more strongly associated with repeat victimization and poor health than any single form of abuse (Ramsey-Klawsnik, 2017; Simmons & Swahnberg, 2021; Williams et al., 2020). Poly-victimization frequently occurs as abusers learn how to be violent from witnessing or personally experiencing violence (Ramsey-Klawsnik, 2017; Simmons & Swahnberg, 2021; Williams et al., 2020). Histories of family violence can perpetuate intergenerational cycles of aggression, where adult children who experienced violence may become perpetrators of elder abuse against their ageing parents (Herrenkohl et al., 2022). Hamby and Grych (2013) have also made the case that such forms of family violence are imbricated in a ‘web’ connected across contexts, over the lifespan from birth to later life, and in the lives of victims, perpetrators, and those involved in violence as both victim and perpetrator. However, there has been limited efforts to systematically study these links resulting in siloed approaches to prevention and intervention.

### Elder abuse and caregiving

Assuming a caregiving role may also be a trigger for the emergence or re-emergence of abuse (Cooper & Livingston, 2020). As their physical and cognitive health declines, older people may develop complex care needs thus increasing their dependency on other family members and/or paid care workers. Increased care loads can exacerbate frustration and burnout in carers resulting in abuse (Cooper & Livingston, 2020). For example, among people with dementia living at home, the prevalence of financial abuse is estimated at 47% (Rogers et al., 2023) with high rates of psychological abuse and physical harm also noted (McCausland et al., 2016). Additionally, linked to their declining health and function, older people themselves may become abusive towards other family members and carers, including older relatives such as spouses, and siblings.

Caring for an older person may also see the re-emergence of family violence and abuse directed towards the older person by other family members such as spouses or adult children (Cooper & Livingston, 2020). For example, spouses or adult children might have been abusive towards the (now) older person when they were younger but then stopped the abusive behavior for a long period of time. However, on becoming a primary caregiver and experiencing growing mutual dependence between them and the older person, the relationship with the older person being cared for may deteriorate and the abuse resume. These dependencies can be intensified by perpetrator risk factors such as substance use disorders, prolonged unemployment, and reliance on the older person for housing, financial assistance, or emotional support (Jackson & Hafemeister, 2015). Patterns of cumulative disadvantage across the life-course can also heighten the risk of elder abuse, especially where poverty, social isolation, or intergenerational conflict persists (Koga et al., 2024).

Research consistently shows that female gender, racial or ethnic minority status, living alone, poor physical health, cognitive impairment, and loneliness are associated with greater vulnerability to abuse (Chen & Dong, 2017; Kong & Easton, 2019; Meyer et al., 2020). According to cumulative inequality and social stress theories, disadvantages in one domain of life reinforce others, accumulating over time and reducing resources and resilience in later life (Dannefer, 2003; Easton & Kong, 2021). For example, women who experience lifelong gendered inequities in employment and caregiving often reach older age with limited income, reduced social networks, and increased dependence on family members for financial or physical support—conditions that elevate their susceptibility to abuse (Dong, 2015). Illuminating these pathways can help response services better understand how a history of victimization might further predispose a person to different types of elder abuse.

### The Australian context

Most life-course studies are quantitative with a dearth of information on the lived experiences of victim survivors. Additionally, they are predominantly conducted in North America with a handful in Asia and Europe. Although around 15% of Australians aged 65 years and over living in the community report experiencing elder abuse within the past 12 months (Qu et al., 2021), to date there have been no Australian studies that have examined elder abuse from a life-course perspective. Yet, Australia has distinctive legislative and socio-cultural features impacting elder abuse. Legislatively, it does not have universal mandatory reporting for elder abuse; reporting obligations apply only in limited circumstances such as abuse perpetrated within formal care relationships (e.g., by aged care staff), where there is a serious and imminent risk to safety triggering professional duty-of-care requirements (e.g., medical practitioners reporting to police or relevant authorities), or where the older person lacks decision-making capacity to act on their own behalf. This approach prioritizes older people’s agency and right to decline intervention (Seniors Rights Victoria, 2018), but also increases their risk of being overlooked by systems and services (The Public Advocate in Queensland & Elder Law Section of the Queensland Law Society, 2022).

Socio-culturally, Australia is a wealthy, politically stable immigrant nation underpinned by British settler-colonial history and the heteronormative logics of white patriarchal ideas (Simic et al., 2025). Up until the 1980s family violence focused mainly on wife-beating among women of reproductive age, child abuse, and incest (Simic et al., 2025). Elder abuse has only been considered a form of family violence since the late 2010s and even then, has received significantly less attention and resources than other forms of family violence. For example, the Federal government allocated A$66 million over five years to implement Australia’s first National Plan to Respond to the Abuse of Older Australians (Elder Abuse) 2019–2023 (McEwan et al., 2024) compared to A$328 million over a similar period (2019–2022) for the National Plan to Reduce Violence Against Women and their Children (Treasurer of the Commonwealth of Australia, 2019). Despite being available in draft form since 2024, the second national plan on elder abuse was only officially released in 2026 and the funding allocated to it remains unclear (Standing Council of Attorneys-General, 2024), while over A$4 billion has been committed since 2022 to end gender-based violence (Budget 2025–26, 2025).

These comparisons are not intended to polarize family violence and elder abuse, but to underscore that as experiences of family violence can extend into later life, harms may be exacerbated for older women yet help-seeking pathways are significantly under-resourced (Brijnath et al., 2026). Documenting the cumulative impact of such violence in the own words of victim-survivors is a powerful and inclusive way to advocate for policy and cultural shifts necessary to change such violent life trajectories. Thus, to address these evidence gaps, we conducted semi-structured interviews with older women in Australia to examine experiences of elder abuse through a life-course lens. This approach enabled us to situate participants’ narratives within both their immediate circumstances and the wider social factors affecting their lives’ adversity and relationship changes.

## Research design and methods

### Design

This study adopted a qualitative descriptive design (Doyle et al., 2020) and drew on data from individual semi-structured interviews with women participating in the Mater-University of Queensland Study of Pregnancy (MUSP) (Najman et al., 2015). The MUSP is a large-scale longitudinal cohort study that began in 1981, enrolling more than 6,700 women during pregnancy (average age 25 years at recruitment), with multiple follow-up assessments conducted over the subsequent four decades (Najman et al., 2015).

### Recruitment

Women over the age of 60 who were part of the original MUSP cohort (recruited 1981–1983) were considered eligible if they had taken part in the most recent wave of data collection, reported experiencing abuse in the past 12 months, and indicated willingness to be interviewed following survey completion. These participants were identified through survey responses collected between October 2023 and November 2024. Among 1,428 respondents, 224 described their experiences of abuse as either ‘somewhat serious’ or ‘very serious,’ and these cases were flagged for further review by S.M. From this group, women reporting either multiple types of abuse (e.g., financial plus emotional) or three or more distinct abusive acts (e.g., theft, withholding of funds, coercion to sign documents) were prioritized, resulting in a shortlist of 93.

B.B. and R.M. reviewed this shortlist to create a purposive sample designed to maximize variation in demographic characteristics (such as socioeconomic status), categories of abuse (physical, emotional/psychological, and financial), and broader contextual circumstances (such as marital status). Guided by this sampling framework (see Supplement 1 in Supplementary Material), 21 women were invited by S.M. to participate. Nineteen agreed, and 17 ultimately took part in interviews. The target was 20 participants but data collection was concluded at 17 when thematic saturation was achieved, with no new insights emerging (Hennink & Kaiser, 2022).

### Procedures

Ethics approval for the project was obtained from the University of Queensland Human Research Ethics Committee (Reference: 2023/HE001159), with additional governance oversight provided by the National Ageing Research Institute. Written or verbally recorded consent was obtained from participants at both the survey and interview stages. All data handling complied with the National Statement on Ethical Conduct in Human Research (section 3.1.45), with storage and analysis carried out within secure systems at the University of Queensland and the National Ageing Research Institute. Only designated data managers and members of the research team were permitted access to raw datasets. To ensure confidentiality, each participant was allocated a study ID, and identifying information was stored separately from the de-identified research data.

Between May and December 2024, R.M. (BA Hons)—a female qualitative researcher with a graduate certificate in Domestic and Family Violence and prior experience in elder abuse research—conducted all interviews by telephone. Each conversation was one-on-one, with no additional people present. At the outset, participants were informed of the interviewer’s role and qualifications. Interviews explored women’s recent experiences of elder abuse, the broader circumstances surrounding those experiences, patterns of help-seeking, interactions with services, and the impact of abuse on personal relationships (see Supplement 2 in Supplementary Materials). The guide used to structure the discussions was not pilot tested, nor were participants given a copy in advance. Interviews were in English, lasted up to 90 minutes, and were digitally recorded, with supplementary notes taken during the interview. Participants were reimbursed for their time with a $50 gift card. No follow-up interviews were conducted.

In cases of current or ongoing abuse a three-step protocol was implemented to ensure participant safety (see Supplement 3 in Supplementary Materials). First, all participants received information about available counselling and support services. Second, where a participant appeared to be at immediate risk, their case was referred to A.C., an experienced practitioner in trauma support for adults. Third, A.C. provided follow-up telephone debriefing and, where appropriate, facilitated referrals into formal support services. Referrals could be triggered either through the routine weekly review of survey data or directly after an interview if concerns were identified. Across the study, one participant required referral following their interview.

### Analysis

Interview recordings were professionally transcribed and checked by R.M. to ensure accuracy and to remove identifying details such as place names, employers, or personal identifiers. Transcripts were not shared back with participants for member checking. Data were thematically analysed (Braun & Clarke, 2006), incorporating inductive and deductive strategies. To begin, R.M. and B.B. independently coded two transcripts, then compared interpretations to establish consistency.

From this process, a joint coding framework was developed and tested on an additional two transcripts to refine reliability and capture the depth of participants’ accounts. Any differences in coding were resolved collaboratively. R.M. subsequently applied the agreed schema to the remaining transcripts using NVivo 14. Draft codes were then organized into initial themes, which were presented to the wider team (J.N., S.M., T.R.M.) for review. Through these discussions, categories were revised, expanded, and reorganized, allowing new themes to be incorporated as analysis progressed.

The analysis was informed by the literature relevant to elder abuse and life-course studies (Awuviry-Newton et al., 2025; Burnes et al., 2019; Easton & Kong, 2021; Herrenkohl et al., 2022; Wiklund et al., 2022). The research team reached agreement on the final analyses through collective discussion and consensus. Deductive codes were informed by the interview guide and prior literature, while inductive codes reflected patterns emerging directly from participants’ accounts. Credibility and trustworthiness were strengthened through the collaborative involvement of several researchers in coding and interpretation, comparison of findings with existing scholarship, careful maintenance of audit trails, and clear documentation of the analytic steps taken to arrive at conclusions (Nowell et al., 2017).

In presenting the results, illustrative quotations are included to highlight participants’ perspectives. This article focuses specifically on themes related to prior abuse, poly victimization, and disadvantage while additional findings such as those concerning help-seeking are reported separately (Brijnath et al., 2026). Throughout this article, the term ‘perpetrator’ is used to denote those who were identified by participants as acting abusively toward them and the term ‘victim’ for those experiencing abuse. The phrase, ‘recent abuse’ is used to refer to incidents occurring within the past year. Participants were not asked to provide feedback on the thematic results. The study followed the Consolidated Criteria for Reporting Qualitative Research (COREQ) (Tong et al., 2007).

## Results

The study included 17 women aged 61 to 76 years (mean 65.8, SD 4.5). All participants were relatively healthy, cognitively well, and independent in daily living. Seven (41%) resided in rural or remote areas, and 13 (76.5%) reported annual incomes below $35,000, reflecting a predominantly low-income sample. To protect confidentiality, only limited socio-demographic details are reported in the excerpts below.

Thematic analysis revealed that experiences of family victimization, such as childhood abuse and intimate partner violence, shaped participants’ susceptibility to and tolerance of abuse in later life. Lifelong exposure to violence also increased vulnerability to poly-victimization, as some participants’ children, having witnessed abuse growing up, reproduced similar behaviors towards their ageing parents. Health-related challenges and economic disadvantage further compounded these experiences, constraining participants’ ability to resist or escape abuse and reinforcing enduring patterns of dependence and control. Many participants commented that their abuse, especially by younger family members, occurred in a broader social context characterized by violent and misogynistic media content, a growing intergenerational wealth gap, and gendered ageism. They reflected that their current circumstances stood in stark contrast to the hopeful and optimistic visions they once held for their lives:

Everything was just so optimistic, and it was going to be wonderful, and I was going to have this child and, you know, we’d all live happily ever after. Yeah, well life’s not like that *(Participant #1).*

So much has changed in my life and virtually none of it has been good. None of it… How did I get here? *(Participant #3)*

### A history of family victimization

In nearly all cases, participants had experienced abuse earlier in their lives, including several who had experienced or witnessed verbal/emotional, physical, or sexual abuse in childhood.

Because of what I’d been through in my life as a child, it [later abuse] was just another fucking thing, just get on with it… When you’re raised a certain way [with abuse], you don’t have these dreams of having this house with the white picket fence. You’re just living moment to moment … You’re hoping to do the best in your situation *(Participant #9).*

Childhood abuse, commonly perpetuated by parents or siblings, often created perceptions in our participants that violence and abuse were a normal part of family dynamics. Many went onto experience family violence in their intimate partner relations.

My first husband, he was a violent drunk and I used to get belted up every weekend… I lived with an abusive father as well… When you’ve experienced so much of it, like I have, you do tend to normalize things, and you tend to let it sort of slide *(Participant #3).*

Negative past experiences of disclosing abuse and violence also often led to participants internalizing their shame and avoiding disclosure as attempts to seek support early on were often dismissed. This silencing was related to historical cultural norms around gender roles and relationship expectations wherein women were socialized to tolerate poor treatment in intimate relationships:

That’s sort of how we grew up… The husband ruled the roost and if you weren’t quick enough to get out of the way, you probably get a clip across the ear *(Participant #6).*

I wanted to confide in somebody, and I started to say something, and my Mum said to me at the time ‘Oh, you made your bed, you lie in it’… I never told anyone anything after that *(Participant #15).*

### Multidimensional forms of poly-victimization

All participants experienced poly-victimization, i.e., they experienced multiple forms of abuse either concurrently or across different periods of their life. They all reported verbal or emotional abuse such as repeated yelling, swearing, belittling, threats of being cut off from perpetrators and family (including grandchildren), or pressure to provide childcare. Additionally, financial abuse was reported by eight participants (47%), physical abuse by six (35%), and sexual abuse by one (3%). Perpetrators included adult children (9, 53% of which 5 were sons and 4 daughters), male partners (3, 18%), male ex-partners (2, 12%), siblings (2, 12% of which one was a brother and the other a sister), granddaughters (2, 12%), other relatives (3, 18% including 1 female), and other individuals such as male friends or neighbors (2, 12%).

In some instances, participants had endured abuse from their perpetrator over the entirety of their relationship, which continued into later life. Many described maintaining these relationships out of love, duty, and/or hope (especially when perpetrators were offspring) and reported positive changes in the perpetrator’s behavior and a halt in the abuse over lengthy periods of time. However, abusive behaviors did re-emerge years later:

We copped it regularly, abuse, yeah, for 15 years, and when she [my daughter] got to around about 30 [years of age], she seemed to sort of come out of it, and this last occasion, that hasn’t happened for, you know, well 12 years or so *(Participant #3).*

Seven out of nine participants abused by their adult children disclosed that when their offspring were younger, they had experienced or witnessed abuse perpetrated by the participants’ intimate partners. Participants reflected that previous exposure to family violence may have prompted abusive behaviors from their own children towards them years later:

Her father could be quite abusive and violent… When her father behaved like that, she saw the power that he had, and has gone on to do that (*Participant #1).*

The father of all my kids, he was abusive. There was domestic violence there and he chose [my son] as the kid he didn’t like… Bullies are often the ones who were bullied, and I would put that down to that *(Participant #12).*

Four women (23%) reported abuse from multiple individuals working together to exert control, inflict harm, and/or exploit them. Financial motivations featured in all four cases. In two instances, participants considered there to be a primary abuser who had enlisted the support of their partners or other family members through manipulation and deceit. In the other two cases, participants felt their perpetrators were co-conspirators.

It went on for an hour and a half of being screamed and yelled at [by my son], and… then his wife joined in… I do feel very much she’s been dragged into this. She doesn’t know the whole story *(Participant #12).*

My husband convinced my daughter to sell his half of the house to her, which he did do, and then he promptly put that money into the poker machines and lost it. So…she owns half the house now… That’s why they get together - together, they can outsmart everybody *(Participant #8).*

These accounts highlight that elder abuse in some families operates as a collective or relational process, not just an individual act, with alliance-building intensifying the victim’s isolation and sense of betrayal.

### Declining health and increasing abuse

Diminishing health was repeatedly associated with increased elder abuse. Five participants experienced serious and protracted illnesses while experiencing elder abuse, which limited their ability to assert themselves and provided a pretext for the perpetrator to approach them under the guise of concern:

That [my surgery] was a contributing factor too to our problems because…he’s always been a bit of a control freak. I let him literally take over…because I was just so wrapped up in myself because I was in so much pain *(Participant #6).*

I was in hospital for 10 days and… he rings checking on how I’m going you know…I thought he was just friendly [until he abused me] *(Participant #5).*

Four participants supported partners through illness and reported that the perpetrator’s poor health intensified abusive behavior:

The difficulty, I think, is once you have a person with cancer, be it the male or the female, it’s like they have a hall pass on everything … He wasn’t great leading up to the radiotherapy *(Participant #17).*

He’s injured. So…the more the more pain he was in, the crankier he got *(Participant #8).*

Ongoing mental health conditions experienced by perpetrators were also linked to elder abuse. Participants often traced perpetrators’ difficulties to earlier life experiences, such as childhood trauma, parental separation, or loss, which were viewed as unresolved and managed through maladaptive behaviors:

He [my son] and his wife lost a baby… he won’t talk to people… [And] his father’s death hit him… he started punching in walls and kicking things and all the rest *(Participant #12).*

These accounts illustrate how health-related vulnerabilities can heighten the risk and severity of elder abuse within families, as declining health—whether in the victim, perpetrator, or other family members—can shift power dynamics and foster dependency. Additionally, unaddressed trauma and poor mental health may lead to maladaptive coping and ultimately abuse. Together, these findings underscore the intertwined nature of health and abuse in later life.

### Economic disadvantage and dependence over the life-course

Financial disadvantage was a recurring theme across the interviews, shaping participant’s vulnerability to abuse and their capacity to respond to it. Those experiencing long-term intimate partner violence commonly reflected that greater financial independence would have enabled them to leave abusive relationships much earlier:

Money would have made a huge, big difference in how I have lived my life… I think my kids would’ve been fine, and I would’ve walked and said, ‘I’m not being yelled, I don’t need this, nor do I deserve this’ *(Participant #8).*

Several participants recounted being kept financially dependent on their partners over many years, because they were prevented from working or they were disadvantaged by inequitable control of assets and resources:

I was forced to leave work before I even got pregnant, and I wasn’t allowed to work while I was married… So, it was hard for some years until I received a permanent job… He could never pay me anything to bring up our children… He had told me when I left that his wish for me was to live in [town] and to do it hard *(Participant #15).*

For some participants, the effects of financial control extended well into later life, influencing their decisions about work, housing, and retirement. Their accounts revealed how economic dependence established earlier in life could accumulate across the decades, leaving older women with limited options, persistent insecurity, and an increased vulnerability to abuse in later years. For example, one participant described how her husband’s longstanding financial abuse and misuse of their marital assets had left her without an adequate retirement fund:

Retirement is just going to be a nightmare for me, I’ll be stuck at home with no money… I just have to work out how the heck I’m going to survive and that kind of thing. So that frightens me… But I’ll find something to do. I’ll get to wash houses or wash floors or something (*Participant #8).*

### Contextualizing ageism and sexism

Viewing violent and misogynistic media content, a growing intergenerational wealth gap, and gendered ageism were perceived as contributors to abuse perpetrated by younger family members such as adult children, nephews, and grandchildren. Perpetrator’s engagement with violent and misogynistic content in some online games, films, and pornography was associated by participants with heightened apathy and aggression, alongside a decline in prosocial values such as empathy and respect.

They [government] need to stop the porn stuff happening … we’re raising technology addicted, incompetent, perverted children because we don’t stop them watching … And who knows what else he’s [my perpetrator is] watching *(Participant #4).*

Some participants also suggested that significant generational differences in attitudes to wealth sharing created abusive conditions. Although most participants had a low income, they described their younger family members as resentful of their assets and their apparent unwillingness to ‘share.’ However, as evidenced from the following quote, sharing often meant entirely relinquishing assets such as the family home to younger family members as it was now their ‘turn’ and older people did not deserve to hold onto this wealth.

[The common] viewpoint at the moment is the old people, the old people they are interfering, they should be giving their wealth to the—all the younger people, ‘You don’t need your nice house, nobody wants to hear what you have to say’ and that’s because you’re a woman and that’s because you’re old *(Participant #2).*

I was referring to the neighbor attacking me. He’s an ice addict and he had a knife, and he threatened to kill me and rape me … He was in his 40s. For three years I tried to have him removed from the house next door to me … I found being an older single woman on my own, you become the target… I think he hated women *(Participant #9)*.

As illustrated by the above quote, ageism and sexism intersected to disenfranchise older women. Their marginalization was further exacerbated when younger people expressed limited understanding and appreciation of the challenges they faced over their lives and overlooked the gendered cultural contexts that older women had grown up and raised their children in. Our participants contrasted the roles expected of younger women in contemporary Australia—as confident, independent, and assertive—with attributes expected of them when they were younger—as accommodating, approachable, and reliant on male family members. Gendered ageism and ahistorical contemporary attitudes were perceived as intensifying ideological divisions between parents and their adult children and fostering frustration and emotional estrangement across generations.

I feel like the younger generation of women, they actually are repulsed by my generation… They want to dismiss us, we’re not tolerated, we’re not important *(Participant #2).*

We’re [our culture is] very youth focused. I do feel you get to an age and a stage and all of a sudden you become invisible. And I know from speaking to other women, you know, like at what point did I, like, cease to exist? At what point did my opinion cease to matter?… You think you raise your kids and, you know, they might have a bit of a rebellious phase, you think that they’re going to outgrow it and then it doesn’t happen *(Participant #1).*

## Discussion and implications

This is the first study in Australia to apply a life-course perspective to elder abuse and one of the few internationally that uses qualitative interviews to illuminate the lived experienced of 17 women who have experienced elder abuse. In doing so, our findings highlight how lifelong patterns of trauma, family violence, gendered economic inequality, and health-related vulnerabilities intersect to shape women’s experiences of abuse in later life. Below we discuss these findings and their implications for policy and practice in Australia and other similar contexts.

Our participants’ experiences not only concord with the evidence but also provide nuance to the growing literature in this area that to date has been primarily cross-sectional, quantitative, and North American-centric. Similar to previous studies, a history of childhood abuse and family violence in midlife is associated with elder abuse in later life (Awuviry-Newton et al., 2025; Easton & Kong, 2021; Herrenkohl et al., 2021; 2022; Kong & Easton, 2019; Wang & Dong, 2019). Women, in particular, who experience lifelong abuse view it as part of their familial relationships and way of life (Roberto & McCann, 2021). Yet as our data show, though the women in our study internalized the shame associated with elder abuse, they also recognized it as problematic, and linked it to historical cultural norms associated with heteronormative white patriarchy that stigmatized female disclosure and help seeking. This is an important insight as it shows that while older women may have been previously socialized to accept abusive relationships, which they may continue to remain in, they do not normalize abuse as part of contemporary family dynamics.

Exposure to a history of violence also led to poly-victimization (Ramsey-Klawsnik, 2017; Simmons & Swahnberg, 2021; Williams et al., 2020). This multidimensional concept includes multiple forms of abuse over the life-course (e.g., physical, psychological, financial), as well as multiple perpetrators at different time points (e.g., abusive spouses in midlife and abusive adult children in later life), and multiple perpetrators at the same time (e.g., adult children and grandchildren concurrently abusing an older person). As perpetrators learn how to be violent from witnessing or suffering from violence in earlier life (Ramsey-Klawsnik, 2017; Simmons & Swahnberg, 2021; Williams et al., 2020), the need to intervene in early life to prevent multiple or single forms of elder abuse in later life is highlighted (Awuviry-Newton et al., 2025). Governments should invest in trauma-informed interventions that begin in childhood.

Aligned with current Australian government expenditure, women, children, and young people need safe, stable living situations where respectful social emotional behaviors and skills are modelled and learned. Intervening in early life could also address ageism and age-related stereotypes; a recent review found that intergenerational programs are the only effective, evidence-based intervention for the primary prevention of elder abuse (Owusu-Addo et al., 2025). Inadequately resourced in government funding is older women’s need to access trauma-informed services, social emotional skill-building programs, and income support that seek to empower them, enable them to live in safe housing, and improve their self-efficacy (Meyer et al., 2020).

Other recent systematic reviews and several other studies also show that family violence over the life-course, including elder abuse, is mediated by gender and physical and mental health (Awuviry-Newton et al., 2025; Meyer et al., 2020). Echoing reports by the participants in our study, the association is multidirectional: for victims, poor health in midlife or later life can increase their reliance on family members for care, including those who might be abusive (Easton & Kong, 2021). For perpetrators, ongoing health issues (such as untreated mental illness, addiction, and trauma) or the emergence of age-related diseases (such as cancer or dementia) can amplify lifelong abusive behaviors (Chen & Dong, 2017). As care is feminized, women typically shoulder much of the workload, often to the detriment of their own health and economic wellbeing. Many discontinue paid work to undertake unpaid care for family members (Cooper & Livingston, 2020). In terms of interventions, health professionals treating unwell older people, or their perpetrators may play a key role here by being cognizant of how these lifelong health and care trajectories can impact the vulnerability of older people. By building trust with older people, identifying those experiencing abuse, helping them through safety planning, and making referrals to other social services such as legal or housing, health professionals can take critical steps to stop elder abuse (Brijnath, Cavuoto, et al., 2025; Wang & Dong, 2019). Frontline services need training and resourcing to undertake safety planning and link older women to legal, housing, and financial supports.

Our participants also described the cumulative effects of continuous economic abuse. Women in our sample experienced lifelong abuse and were restricted from participating in the paid economy leaving them financially dependent on their perpetrators and unable to leave abusive relationships. Currently, about 25 percent of Australian women aged 60 or older live in poverty, compared with about 21 percent in men (Impact Economics and Policy, 2025). The risk rises sharply with age—among women aged 80 and over, more than two in five experience poverty (Impact Economics and Policy, 2025). Single older women are particularly vulnerable, reflecting lifelong gender gaps in pay, superannuation, and unpaid care work (Johnson et al., 2022). This gulf is likely to widen as women tend to live longer than men. Thus, alongside interventions to improve older women’s self-efficacy, housing and financial reforms should address older women’s economic emancipation and wellbeing.

Finally, ageist and sexist media discourses should be publicly challenged. Our participants felt that violent and misogynistic media content, a growing intergenerational wealth divide, and gendered ageism were contextual factors in which their abuse occurred. While we could find no evidence to substantiate participant’s comments about media content, declining intergenerational cohesion within families may be shaped by ageist narratives that persist post the COVID-19 pandemic and by widening inequalities in housing wealth between generations. During the pandemic, Australian public and political discourse frequently portrayed older adults’ lives as less valuable than economic interests, with age sometimes used to justify denying them life-saving medical care (Brijnath, Feldman, et al., 2024). In Australia, many aged care residents were neglected, isolated, or confined to their rooms for months, contributing to the disproportionately high death rates in residential facilities (Brijnath, Feldman, et al., 2024; Gilbert et al., 2023). In the pandemic’s aftermath, these sentiments linger, and have grown in the face of a housing crisis that has seen a sharp decline in home ownership among younger people with a concomitant rise in the value of housing assets, often owned by older people ( Ong Viforj and Phelps, 2023 ). Alongside, stagnating wage growth and a rising cost of living in Australia has contributed to greater reliance of younger people on parental financial support and a growing resentment among younger people that the social contract between generations has fractured (Australian Human Rights Commission, 2021). While this narrative is amplified by media discourse and ageist stereotypes (Australian Human Rights Commission, 2024), the underlying economic pressures confronting younger Australians remain both tangible and deeply consequential. Thus, even though our participants were low-income earners and financially disadvantaged, their abuse was situated in a broader social context that undervalued older people (Australian Human Rights Commission, 2021, 2024).

### Limitations

Our recruitment through the MUSP study resulted in a sample largely composed of Caucasian women residing in Queensland. Because participants had to self-identify as having experienced abuse and provide informed consent, those who took part tended to be relatively younger (mean age = 65.8 years, SD = 4.5) and in reasonable health. This profile means their accounts align closely with findings from the wider literature on intimate partner violence among younger women. As such our life-course analysis is wholly gendered. Future research might consider including men, victims aged 80 years and older, individuals with poorer health, or those reliant on care. Moreover, we caution about making inferences from our qualitative data about the extent to which proximate (e.g., poor health) and distal factors (e.g. childhood abuse) over the life course are associated with elder abuse. Our intent with these qualitative data are to explain, not enumerate these associations; the latter analyses will be completed with the MUSP cohort once all quantitative data has been collected. Finally, while we have not delved into how a history of violence might shape help seeking—we do cover this in another article (Brijnath et al., 2026)—we acknowledge that the two are inextricable.

## Conclusion

This study provides the first Australian evidence on elder abuse using a life-course lens, drawing on the lived experiences of older women. The findings reveal that elder abuse is rarely an isolated event but often includes a historical dimension with family violence beginning in childhood or midlife. Poly-victimization is common, and experiences of abuse are compounded by poor health, advancing age, and entrenched gendered economic disadvantage. Rapid technological change, ageism, and increased intergenerational housing and wealth inequity are contemporary contexts in which abuse occurs, and factors that may be amplifying generational divides and generational relations. A life-course perspective highlights the need for prevention and policy responses that address these cumulative, structural, and relational determinants of vulnerability across the lifespan.

## Supplementary Material

gnag122_Supplementary_Data

## Data Availability

The data that support the findings of this study are available on request from the corresponding author (B.B.) or the study lead (J.N.). The data are not publicly available due to IRB restrictions as they contain information that could compromise the privacy of research participants. This study was not preregistered.
