# Supplementary material for: “How did I get here?”: a qualitative life-course analysis of Australian women’s experiences of elder abuse
Source: Gerontologist. 2026 Jun 5;66(7):gnag122. doi: 10.1093/geront/gnag122 (PMC13316421; doi:10.1093/geront/gnag122)
Supplement: gnag122_Supplementary_Data [file gnag122_supplementary_data.pdf]

“How did I get here?”: A qualitative life-course analysis of Australian women’s experiences of elder abuse: Supplementary materials

Bianca Brijnath*, Rachel Muoio, Jake Najman, Shelby Marrington, Alexandra Clavarino, Tara Renae McGee, and Anna Gilbard.

- **Supplement 1: Sampling framework**
- **Supplement 2: Interview guide**
- **Supplement 3: Three-point risk mitigation protocol for participants’ wellbeing**
- **Supplement 4: Consolidated criteria for reporting qualitative studies (COREQ): 32-item checklist**

Supplement 1: Sampling framework

**Vary by…**	**Segment (relates to 12mo prior to survey)**	**Aim**
Group A: Older women are victims of elder abuse		10-12 total
**1. Type of abuse experienced.**	a. Financial	3-4
	b. Emotional/ verbal/ psychological	3-4
	c. Physical	2-3
**2. Relationship to the perpetrator.**	a. Perpetrator is adult child	6-8
	b. Perpetrator is a care provider	3-4
	c. Perpetrator is partner	1-2
	d. Perpetrator is other than a-c	1-2
**3. Receiving care or not**	a. Receiving care	6-8
	b. Not receiving care	4-6
**4. Other factors**	Location, living arrangement, employment, income	A mix
Group B: Older women who have perpetrated (and may also be victims of) elder abuse		5-7 total
**1. Type of abuse perpetrated**	a. Emotional/verbal/psychological	4-6
	b. Physical	0-2
**2. Relationship to the victim (who they provide care for)**	a. Victim is adult child	2-3
	c. Victim is spouse	1-2
	d. Victim is other than a-c	0-2
**3. Other factors.**	Location, living arrangement, employment, income, substances	A mix

Supplement 2: Interview guide

1. Introduction, consent to start recording, and warm-up.	
2. Short life history	What would you say are some key events or changes in your life?<br>How has your family life / relationships (marital or intimate) changed?<br>How has your employment situation changed?<br>How have your relationships with your children changed?
2. Type, range, and duration of abuse experienced	Could you please tell me about the elder abuse that you have experienced?<br>What sort of behaviours were involved? [Probe for other types of abuse].<br>When did this start?<br>How long did this go on for?<br>Are you experiencing elder abuse currently?
3. Characteristics influencing elder abuse experienced	What factors do you think may have contributed/are contributing to [the perpetrators] behaviour? [Probe alcohol or drug abuse; mental health issues; past history of behaviour].<br><br>What was your relationship like with [perpetrator] prior to their abusive behaviour?<br>Was there a particular turning point in your relationship when this behaviour began? Can you describe this?<br>How would you describe your social or support network at the time that you were experiencing abuse?<br>Do you feel that you had a support network you could confide in or receive help from? [Probe: Family, friends, community engagement, service systems].<br>Are there any factors you would say that might have made you particularly vulnerable to elder abuse? [Probe: Economic hardship; change in health].
4. Abuse perpetrated by participant *(for perpetrator sample only)*	Have you ever behaved in ways that could be considered abusive? [Provide examples].<br><br>[Ask all questions in sections 1-2 again in context of abuse perpetrated]:<br>What sort of behaviours were involved?<br>When did this start? How long did this go on for?<br>What was your relationship like with [victim] prior to your [abusive behaviour]? Was there a turning point?<br>Do you feel that you had a support network you could confide in or receive help from?<br>Are there any factors you would say that might have influenced your [abusive behaviour]?
4. Thank you, offer resources and support, close.	

Supplement 3: Three-point risk mitigation protocol for participants' wellbeing

To mitigate risk of distress or discomfort, staff will:

- Take care to monitor participants' wellbeing by remaining vigilant to identify signs of distress or any other adverse events that may affect participants or staff.
- Interviews will be conducted by RM (BA Hons), an experienced qualitative researcher trained in trauma-informed practice as per a Graduate Certificate in Domestic and Family Violence.
- All participants will be offered time to break from the sessions, and may request a break at any time.

If a participant is feeling upset, distressed or at risk of harm, staff will:

- Offer the participant a break and provide basic comfort and assistance in calming down the upset or distressed participant. Basic comfort includes actions such as offering a glass of water and a tissue (or suggesting these actions if the session is conducted remotely).
- Offer to contact a family member or friend for the participant.
- Remind the participant that their participation is voluntary and that they will face no negative consequences if they choose to withdraw their participation; and ask the participant if they would like to continue or withdraw their participation.
- Offer to put the participant in contact with the project investigator, AC, for debriefing over the phone. AC is a social worker who has extensive clinical experience working with adults suffering from trauma.
- Encourage the participant to seek counselling or other mental health support through their healthcare provider and/or Employee Assistance Program, and/or offer the following resources to seek further emotional support or counselling:
    - Lifeline: 13 11 14 (available 24/7) or Lifeline crisis support online chat at www.lifeline.org.au (available 7pm to midnight, 7 days per week).
    - Beyond Blue: 1300 22 4636 (available 24/7) or Beyond Blue online chat at www.beyondblue.org.au (available 3pm until midnight, 7 days per week).
    - 1800 ELDERHelp: 1800 353 374 (automatically redirects callers seeking information and advice on elder abuse with existing phone line service in their jurisdiction).
    - Further resources as per Table 1 ('Counselling and Support Contact Information' prepared by the University of Queensland).
    - Researchers will record any adverse events in an incident log and report any adverse events as soon as practicable to the project investigator, AC.
- Record any adverse events in an incident log and report any adverse events as soon as practicable to the project investigator, AC.

Table 1: Counselling and Support Contact Information

If you need immediate help, call 000

Service name	Contact information	Service description
**Beyond Blue**	P: 1300 22 4636<br>W: www.beyondblue.org.au	Beyond Blue provides information and support to help everyone in Australia achieve their best possible mental health, whatever their age and wherever they live.
**Lifeline**	P: 13 11 14<br>Text: 0477 13 11 14 (6pm to 12pm AEST)<br>W: www.lifeline.org.au	Lifeline is a national charity providing all Australians experiencing a personal crisis with access to 24 hour crisis support and suicide prevention services.
**ELDERHelp**	P: 1800 353 374<br>W: humanrights.gov.au/elder abuse	A free call that automatically redirects callers seeking information and advice on elder abuse with the phone service in their state or territory.
**1800RESPECT**	P: 1300 737 732<br>www.1800respect.org.au	Healthy relationships start with feeling respected and safe. You have a right to respect and safety in all your relationships. If you are worried about unhealthy, abusive or violent behaviour in any of your relationships.
**Relationships Australia Elder Abuse Prevention and Support Service**	P: 1300 364 277 (M-F 8am to 8pm, Sat 9am to 5pm AEST)<br>W: www.relationships.org.au	In Queensland, the Elder Abuse Prevention and Support Service is a Case Managed service for people 60+ (or 50+ for Aboriginal or Torres Strait Islander people) who are at risk or are experiencing Elder Abuse.
**Carer Gateway**	P: 1800 422 737<br>W: www.carergateway.gov.au	Connect with an Australia-wide network of carer service providers and support for carers.
**MyAgedCare**	P: 1800 200 422<br>W: www.myagedcare.gov.au	For assistance with learning what services are available, assessments for eligibility, location of service providers.
**Blue Knot Foundation**	P: 1300 657 380<br>E: helpline@blueknot.org.au<br>W: www.blueknot.org.au	The helpline has trained, experienced and trauma-informed counsellors to support adult survivors of childhood trauma and abuse. Monday – Sunday between 9am - 5pm AEST
**Suicide Call Back Service**	P: 1300 659 467<br>W: www.suicidecallbackservice.org.au	National services that provides free 24/7 phone, video and online professional counselling to people who are affected by suicide.
**Alcohol and Drug Foundation**	P: 1300 858 584<br>E: druginfo@adf.org.au<br>W: www.adf.org.au	Looking for non-judgmental and confidential information or advice? DrugInfo can help. Are you seeking help for yourself or are you concerned about a loved one? Call or email DrugInfo for facts about alcohol and other drugs, advice on how to support a loved one, and to get connected with relevant services in your state and territory.
**Arafmi**	P: 1300 554 660<br>W: www.arafmi.com.au	Arafmi works to enhance the wellbeing of people with mental illness, their families and carers by providing quality support, accommodation, education and advocacy services.
**13YARN**	P: 13 92 76<br>W: www.13yarn.org.au	13YARN is the first national crisis support line for mob who are feeling overwhelmed or

		having difficulty coping. We offer a confidential one-on-one yarning opportunity with a Lifeline-trained Aboriginal & Torres Strait Islander Crisis Supporter who can provide crisis support 24 hours a day, 7 days a week.
**Transcultural Mental Health Services**	Qld: 1800 188 189<br>NSW: 1800 011 511 (24/7)<br>Australia wide: P: 131 450	Specialist services that work to ensure people from culturally and linguistically diverse (CALD) backgrounds receive culturally responsive mental health care and support and interpreting services.

Supplement 4: Consolidated criteria for reporting qualitative studies (COREQ): 32-item checklist

No	Item	Guide questions/description	Section
**Domain 1: Research team and reflexivity**			
Personal Characteristics			Section
1.	Interviewer/ facilitator	Which author/s conducted the interview or focus group?	Methods (Procedure)
2.	Credentials	What were the researcher's credentials? *E.g. PhD, MD*	Methods (Procedure)
3.	Occupation	What was their occupation at the time of the study?	Methods (Procedure)
4.	Gender	Was the researcher male or female?	Methods (Procedure)
5.	Experience and training	What experience or training did the researcher have?	Methods (Procedure)
Relationship with participants			
6.	Relationship established	Was a relationship established prior to study commencement?	Methods (Design and Recruitment)
7.	Participant knowledge of the interviewer	What did the participants know about the researcher? e.*g. personal goals, reasons for doing the research.*	Methods (Procedure)
8.	Interviewer characteristics	What characteristics were reported about the interviewer/facilitator? e.g. *Bias, assumptions, reasons and interests in the research topic.*	Methods (Procedure)
**Domain 2: study design**			
Theoretical framework			

No	Item	Guide questions/description	Section
9.	Methodological orientation and Theory	What methodological orientation was stated to underpin the study? *e.g. grounded theory, discourse analysis, ethnography, phenomenology, content analysis.*	Methods (Design); Methods (Analysis)
Participant selection			
10.	Sampling	How were participants selected? *e.g. purposive, convenience, consecutive, snowball*	Methods (Recruitment)
11.	Method of approach	How were participants approached? e.*g. face-to-face, telephone, mail, email*	Methods (Recruitment)
12.	Sample size	How many participants were in the study?	Methods (Recruitment)
13.	Non-participation	How many people refused to participate or dropped out? Reasons?	Methods (Recruitment)
Setting			
14.	Setting of data collection	Where was the data collected? e.*g. home, clinic, workplace*	Methods (Procedure)
15.	Presence of non-participants	Was anyone else present besides the participants and researchers?	Methods (Procedure)
16.	Description of sample	What are the important characteristics of the sample? *e.g. demographic data, date*	Methods (Procedure); Results
Data collection			
17.	Interview guide	Were questions, prompts, guides provided by the authors?<br>Was it pilot tested?	Methods (Procedure); Supplement 2.
18.	Repeat interviews	Were repeat interviews carried out? If yes, how many?	Methods (Procedure)
19.	Audio/visual recording	Did the research use audio or visual recording to collect the data?	Methods (Procedure)

No	Item	Guide questions/description	Section
20.	Field notes	Were field notes made during and/or after the interview or focus group?	Methods (Procedure)
21.	Duration	What was the duration of the interviews or focus group?	Methods (Procedure)
22.	Data saturation	Was data saturation discussed?	Methods (Recruitment)
23.	Transcripts returned	Were transcripts returned to participants for comment and/or correction?	Methods (Analysis)
**Domain 3: analysis and findings**			
Data analysis			
24.	Number of data coders	How many data coders coded the data?	Methods (Analysis)
25.	Description of the coding tree	Did authors provide a description of the coding tree?	Not included
26.	Derivation of themes	Were themes identified in advance or derived from the data?	Methods (Analysis)
27.	Software	What software, if applicable, was used to manage the data?	Methods (Analysis)
28.	Participant checking	Did participants provide feedback on the findings?	Methods (Analysis)
Reporting			
29.	Quotations presented	Were participant quotations presented to illustrate the themes / findings? Was each quotation identified? e.*g. participant number*	Results
30.	Data and findings consistent	Was there consistency between the data presented and the findings?	Methods (Analysis)
31.	Clarity of major themes	Were major themes clearly presented in the findings?	Results

No	Item	Guide questions/description	Section
32.	Clarity of minor themes	Is there a description of diverse cases or discussion of minor themes?	Results
